# Analysis of Fatty Acid Compositions and Acid Values of Krill Oil Supplementary Products from the Korean Market

**DOI:** 10.4014/jmb.2406.06026

**Published:** 2024-08-23

**Authors:** Ji Yun Lee, Jun-Bae Hong, Bo-Kyung Kim, Seong Bo Shim, Hae Won Jang, Jung-Bin Lee

**Affiliations:** 1 Department of Food Science and Biotechnology, Sungshin Women’s University, 55, 76 ga-gil, Gangbuk-gu, Seoul, 01133, Republic of Korea; 2 Korea Consumer A gency, 54 Yongdu-ro, Maengdong-myeon, Eumseong-gun, Chungcheongbuk-do, 27738, Republic of Korea

**Keywords:** Fatty acids, acid value, krill oil, krill oil supplement, gas chromatograph

## Abstract

In order to provide the qualitative data for the 20 commercially available krill oil supplementary products, the levels of omega-3 polyunsaturated fatty acids (PUFA) such as docosahexaenoic acid (DHA) and eicosapentaenoic acid (EPA), fatty acid compositions, and chemical indices, including acid values, of the supplements, were determined. The acid values ranged from 7.4 to 43.7 mg of potassium hydroxide (KOH)/ g of oil. The relative percentages of EPA and DHA in the oils ranged from 14.2 to 34.8 % (w/w). Although all 20 krill oil supplements used 100% krill oil as raw material, the fatty acid composition of 4 samples differed from typical krill oil in terms of the content of myristic acid (C14:0), palmitic acid (C16:0), palmitoleic acid (C16:1), linoleic acid (C18:2, n-6), and eicosenoic acid (C20:1, n-9). Accordingly, the Ministry of Food and Drug Safety recently standardized linoleic acid (3% or less) and myristic acid (5–13%) as part of the fatty acid components of krill oil. This study provides a reference for analyzing the chemical and nutritional properties and evaluating the adulteration of krill oil supplements in the Korean market.

## Introduction

Antarctic krill (*Euphausia superba*) are small, abundant crustaceans native to the Southern Ocean around Antarctica that are processed to produce krill meal for agriculture and aquaculture feeds and krill oil for humans [1-3]. Fish stocks have been into decline resulted from overfishing and environmental pollution in recent years, whereas krill could be an alternative marine resource [4]. Commission for the Conservation of Antarctic Marine Living Resources (CCAMLR) has regulated ‘trigger’ level within 620,000 tons across four regions in the southwest Atlantic per year to ensure the sustainability of krill fishery activity [5]. Nevertheless, the current annual catch is approximately 370,000 tons, suggesting that there is a significant underutilizing of krill without expansion in the krill fishery [5].

Marine n-3 PUFA, including EPA and DHA, have been demonstrated to play beneficial roles in the prevention of cardiovascular disease (CVD) [6, 7]. In addition to decreasing circulating lipid concentrations [8], n-3 PUFA have been found to reduce systolic blood pressure, inflammatory processes, and platelet aggregation, as well as prevent arrhythmias [6, 9]. Consumption of n-3 PUFA, including EPA and DHA, occurs mainly through intake of oily fish or through supplements, where the n-3 PUFA are usually esterified to triglyceride glycerol (TG). However, other marine sources, such as krill oil, in which the n-3 PUFA are esterified to phospholipid glycerol (PL), can also be used as sources for n-3 PUFA. Krill oil is valued as a dietary supplement due to its high concentrations of long-chain (≥ C_20_) n-3 PUFA: EPA (13.8-20.3%) and DHA (5.6-17.4%) [10]. Consumption of n-3 PUFA esterified to PLs has been shown to exert a wide range of health benefits, such as plasma lipid reduction [11], enhanced bioavailability of EPA and DHA [12-14], as well as anti-inflammatory [15], muscle mass or muscular strength [16] and antioxidant activity [17]. Based on these compositions, the development of functional food has led to a steady increase in consumer demand for long-chain n-3 containing oils, mainly in better absorbance in krill than fish [18, 19]. According to a previous study, edible insect oils have shown similar component analysis to vegetable oil regarding fatty acid profiles, and krill and fish oils also have similar fatty acid profiles [20]. Furthermore, krill oil has thus been receiving increasing attention due to its nutritional and functional potential as a substitute for fish oil.

Although studies on oil, including the lipid content of Antarctic krill [3], fatty acid profiles of microencapsulated krill oil [21], free fatty acid analysis of commercial krill oils in New Zealand [22], the relationship between phospholipid content and a sum of DHA and EPA in krill oil [23], detection of toxic α-dicarbonyl compounds in commercial sesame and perilla oil [24], have been conducted, there were no studies comparing quality characteristics about commercial krill oil supplements in Korea. Also, there are lots of krill oil products that advertise their biochemical functionality with no authorized marks, which are well misunderstood as a functional food to consumers. Nonetheless, there is a lack of comprehensive reviews on the quality and safety aspects of krill oil products. To our knowledge, only limited studies have been conducted to measure the safety and chemical and nutritional properties of commercial krill oil supplementary products; therefore, this study aimed to analyze the chemical and nutritional properties of 20 commercially available krill oil supplements in the Korean market.

## Materials and Methods

### Materials

Twenty krill oil supplements (KO) were obtained from an online shopping mall in February 2020. Each was carefully selected based on its market share and manufacturer, namely, the top 20 products based on market share and products that were readily available via common distribution channels. All chemicals used were of analytical grade. Diethyl ether, ethanol, methanol, and iso-octane were obtained from J.T. Baker (J.T. Baker Inc., USA). The 14% boron trifluoride (BF3) and the 37 Component fatty acid methyl esters (FAME) Mix were obtained from Supelco (Supelco, Inc., USA). Potassium hydroxide ethanolic solutions (0.1 N) and sodium chloride were obtained from Duksan (Duksan General Science, Republic of Korea).

### Sample Preparation

A total of 20 krill oil products were included in this study and KOs were classified as numbered backward randomly from 1 to 20. Upon the arrival of the krill oil supplements from the online shopping mall, they were separated into their respective oil and capsule portions. The capsule portions of the products were discarded, and the oil portions were transferred into a small glass vial, flushed with nitrogen, and maintained at -18°C until they were analyzed for acid value (AV) and fatty acid composition. The samples were prepared for triplicate measurements of AV and fatty acid composition analysis.

### Acid Value (AV) Determination

The autoxidation of krill oils is the prominent cause of quality deterioration. AV was selected to measure the storage-related oil stability and deterioration of the oils. The AV determination method was slightly modified from the Korean Food Code and AOCS Cd 3d-63 methods [23]. Briefly, potassium hydroxide ethanolic solutions (0.1 N) were prepared, and 5.0 g of krill oil were individually dissolved in diethyl ether: ethanol (1:1, v/v). The AV values were determined at the visual end-point using phenolphthalein.

### Fatty Acids Analysis

The FAME of the krill oils were prepared according to the Korean Food Code [25] and modified method according to previous research [26, 27]. Approximately 0.25 g of each oil was weighed into a glass tube. After that, 1.5 ml of 0.5 N methanolic sodium hydroxide was added, and the mixture was placed in boiling water for 5 min. Following this, the tubes were cooled, and 2.0 ml of 14% BF3 was added and boiled for 30 min. After this, the tubes were cooled, and 1.0 ml of iso-octane and 5.0 ml of saturated sodium chloride (NaCl) solution were added. The tubes were then shaken vigorously and left for 10 min. The upper layer was then transferred into a small vial and stored at 0°C until the time of analysis.

The fatty acid compositions were determined using a gas chromatograph (Shimadzu, GC 2010 Plus, Japan) equipped with a flame ionization detector (FID) and an SP-2560 (0.25 mm ID, 100 m long, 0.2 μm film thickness, Supelco, Inc.). The temperature program started at 140°C for 5 min, increased to 220°C at a rate of 5°C/min and then increased to 240°C at a rate of 5°C/min and held at this temperature for 30 min. The injector and detector temperatures were both set at 260°C. Nitrogen was used as a carrier gas, and the split ratio was set at 1:100. The FAME was identified by comparing the retention times of the samples to those of standard samples (Supelco 37 Component FAME Mix).

## Results and Discussion

### Acid Values (AVs) of the Krill Oil Supplements

The AV is implemented as a reliable indicator of the extent of free fatty acids formation due to lipase-mediated hydrolysis, which contributes to the occurrence of hydrolytic rancidity in oils [28]. The measurement of AV allows for the direct transformation of AV into a percentage of fatty acids, assuming the absence of other acids in the oil, as outlined in the AOCS method Cd 3d-63 [29]. The AVs of the 20 commercial krill oils analyzed in this study are shown in Table 1. The AVs of the commercially available krill oil products examined in this study varied between 7.4 and 43.7 mg KOH/ g of krill oil. Among the 20 krill oil supplements, 65% of the samples exhibited values exceeding 20 mg KOH/ g which showed similar results in a previous study assessing the stability of phospholipids-rich Antarctic krill oil. In a previous study, the krill oil samples were stored in various conditions (temperature, exposure to oxygen, and presence of light) and the results demonstrated that the AV exhibited an increase at 40°C, with oxygen exerting a more pronounced influence on the hydrolysis of krill oil in comparison to light. The AVs of whole samples were analyzed not exceeding 30 mg KOH/ g but over approximately 20 mg KOH/ g [30]. However, in this study, KO 7 showed the highest AV of 43.7 mg KOH/ g, whereas KO 13 showed the lowest AV of 7.4 mg KOH/ g. Some countries have set a maximum limit for AV about specific types of fish or other marine oils as follows: AV limit of 0.5 mg KOH/ g set by British and European Pharmacopeia; 2.0 mg KOH/ g set by the Australian government; 3 mg KOH/ g set by the US Pharmacopeia and Codex [28]. Notably, the maximum allowable AV for fish oil with a high phospholipid concentration of 30% or more like krill oil is reported as 45 mg KOH/ g from the Codex Standard [31]. The AVs of the commercially available krill oil supplements included in this study were therefore within the range of the maximum allowable AV level according to the international standard.

### Fatty Acids Analysis

It is widely reported that the n-3 PUFAs derived from dietary lipids, mainly EPA and DHA, play a significant role in human health [32-34]. Krill oils are rich in EPA and DHA, and they are consumed as dietary supplements for n-3 PUFAs [19]. The results of the fatty acid analysis are shown in Tables 2-1 and 2-2. The EPA content of the commercial krill oil supplement samples ranged from 9.7 to 26.0% (w/w); the DHA content ranged from 4.5 to 11.1 % (w/w); and the total omega-3 fatty acid content ranged from 14.2 to 34.8% (w/w). While intake of supplemental krill oil products is known to decrease the risk of cardiovascular diseases, the supplementary intake of omega-3 fatty acids has strict guidelines on its dose due to the increased risk of bleeding [35]. According to the European Food Safety Authority [35], the recommended intake for EPA and DHA is up to 5 g/ day up to 16 weeks, and supplementary intake of DHA by itself is 2-4 g/ day without supervision by a health care provider. The krill oil supplementary products tested in this study were therefore within the range of the regulatory safety guidelines. The American Heart Association's dietary guidelines recommend consuming two servings of fatty fish per week for the primary prevention of coronary diseases [36]. This recommendation equates to an intake of approximately 250-500 mg of EPA and DHA per day [37]. A previous study has shown that the daily consumption of 3 g of krill oil, which contains 543 mg of EPA+DHA, leads to a comparable elevation in plasma EPA and DHA levels as the ingestion of fish oil containing 864 mg of EPA+DHA [14]. In this study, the krill oil KO3, which contained the highest amount of EPA+DHA, approximately 520 mg per 1.5 g of krill oil, exhibited similar results to the previous research. Furthermore, the results of the fatty acid analysis in this study were generally lower than those for omega-3 supplements (fish oils), according to a previous study, which reported that the EPA content of commercial fish oil supplement samples ranged from 5.11 to 59.16% (w/w), the DHA content ranged from 10.87 to 73.91% (w/w), and the total omega-3 fatty acid content ranged from 29.26 to 87.43% (w/w) [38].

Especially, four commercial krill oils (KO 2, 6, 8, and 15) showed high levels (31.4, 29.6, 29.9, and 30.0% (w/w), respectively) of linoleic acid (C18:2, n-6) in Table 2-2. The palmitic acid (C16:0) content of the aforementioned four KOs was 11.2, 10.8, 10.7, and 10.5% (w/w), respectively, and the palmitoleic acid (C16:1) content ranged from 0.2 to 0.3% (w/w) (Table 2-1). In addition, myristic acid (C14:0) was not detected in these four KOs. Although all 20 samples used 100% krill oil as a raw material, the fatty acid composition of the four aforementioned samples was different from typical krill oil, particularly in their linoleic acid (C18:2, n-6), palmitic acid (C16:0), palmitoleic acid (C16:1), and myristic acid (C14:0) contents. These results present strong evidence that other vegetable oils and fats have been incorporated into these krill oils because their fatty acid compositions differ from commercial krill oil. Similar fatty acid compositions to those defined by the Codex Standard [31] were reported in previous studies [13, 39]. Moreover, soybean phospholipids are more affordable and easier to obtain than krill phospholipids, leading to frequent adulteration of krill phospholipids with soybean phospholipids in cases of food fraud or adulteration [40].

The main fatty acids present in krill oil are C14:0, C16:0, C16:1, C18:1, C20:1, C20:5 (EPA), and C22:6 (DHA)[41-43]. The typical fatty acid composition of twenty krill oil supplements, krill oil [29], in comparison with soybean oil and arachis oil [44], is shown in Table 3. According to the Codex Standards [31], the fatty acid composition of krill oil should be as follows: linoleic acid (C18:2, n-6), ND-3.0% (w/w); palmitic acid (C16:0), 17.0-24.6% (w/w); palmitoleic acid (C16:1), 2.5-9.0% (w/w); and myristic acid (C14:0), 5.0-13.0% (w/w). In this study, the content of linoleic acid (C18:2, n-6) in sixteen krill oils ranged from 0.5 to 2.8% (w/w), the palmitic acid (C16:0) content ranged from 17.0 to 23.8% (w/w), the palmitoleic acid (C16:1) content ranged from 4.5 to 10.6%(w/w), and the myristic acid (C14:0) content ranged from 6.8 to 12.0% (w/w). Of the twenty krill oil supplements examined, sixteen met the Codex Standard for krill oil proximately, while the remaining four (KO 2, 6, 8, and 15) did not meet the criteria. According to the Codex Standards of soybean and arachis oil (peanut oil), four krill oils had similar tendencies in terms of myristic acid (C14:0), palmitic acid (C16:0), palmitoleic acid (C16:1), linoleic acid (C18:2, n-6), and eicosenoic acid (C20:1, n-9). Especially, the ranges of myristic acid (C14:0), palmitic acid (C16:0), palmitoleic acid (C16:1), and linoleic acid (C18:2, n-6) showed common with soybean and arachis oil. In soybean phospholipid samples, the fatty acid composition exhibited great differences between linolenic acid (C18:3, n-6) and linoleic acid (C18:2, n-6) compared with the krill phospholipid samples in a previous study. In detail, palmitic acid (C16:0), linolenic acid (C18:3, n-6), linoleic acid (C18:2, n-6), and oleic acid (C18:1) were the major fatty acids in soybean phospholipids, which had similarity, except for linolenic acid (C18:3, n-6), with four krill oils (KO 2, 6, 8, and 15) in this study [40]. Also, the presence of a high percentage value of eicosenoic acid (C20:1, n-9) in other oils such as olive oil could be a concern with adulteration with peanut oil [45]. Cultivars from Argentina, Bolivia, Poland, and USA have presented similarly with four krill oils (KO 2, 6, 8, and 15) in this study as follows: palmitic acid (C16:0), 10.2–13.0% (w/w); linoleic acid (C18:2, n-6), 24.1–35.3% (w/w); eicosenoic acid (C20:1, n-9), 0.7–1.7% (w/w) in arachis oil [46, 47]. Previously, some fish oil supplements showed high eicosapentaenoic acid (C20:5, n-3), and were obtained with high AV over 1.00 mg KOH/ g [38]. This result was identified similarly in KO 7 containing high eicosapentaenoic acid (C20:5, n-3) with higher AV than other KOs. Otherwise, KO 13 showed the lowest AV but high content of palmitoleic acid (C16:1) and oleic acid (C18:1). In previous vegetable seeds oil research, groundnut oil only presented palmitoleic acid (C16:1) and similar content of oleic acid (C18:1) with soybean oil but showed the lowest AV in comparison with other vegetable seed oils [48].

**Fig. 1:**
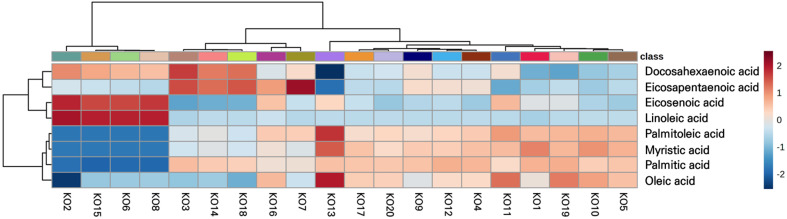
Heatmap hierarchical clustering analysis (heatmap HCA) of fatty acid profiles in 20 krill oil supplements using major fatty acids according to Codex Standard 329-2017 (2017). KO indicates krill oil and 20 krill oil supplements are classified as numbered backward randomly. Fatty acid concentrations (g/100 g; % (w/w)) represent the mean of triplicate experiments displayed in the heatmap. The heatmap was generated by merging three transcript lists from each sample.

To identify the variable distribution among commercial krill oil supplements, hierarchical clustering analysis (HCA) of the fatty acid profiles of the tested twenty oils was employed, the results of which are presented as a visual heat map based on the Euclidean correlation (Fig. 1). The A heatmap was examined to investigate similarities and differences between the samples of fatty acid profiles. The color index next to the fatty acid class indicates the relative concentrations of the fatty acids, from high (dark red) to low (dark blue).

As shown in Fig. 1, it can be observed that KO 2, 15, 6, and 8, suspected of being contaminated with vegetable oils, showed significant differences based on five fatty acids. In detail, a suspicious group (KO 2, 15, 6, and 8) contained lower contents of palmitoleic acid (C16:1), myristic acid (C14:0), and palmitic acid (C16:0), whereas higher contents of linoleic acid (C18:2, n-6) and eicosenoic acid (C20:1, n-9) than other KOs. These results were in line with Table 3 according to the Codex Standards for krill, soybean, and arachis oil [31, 44]. Therefore, in krill oil supplements, palmitoleic acid, myristic acid, palmitic acid, linoleic acid, and eicosenoic acid could be indicators of contamination with vegetable oils.

## Conclusion

This study supplies quality characteristics of twenty krill oil supplements from an online shopping mall in Korea. The range of AV among twenty krill oil supplements detected from 7.4 to 43.7 mg KOH/ g and thirteen samples exceeded 20 mg KOH/ g nevertheless these results conformed to the notified Codex Standard (45 mg KOH/ g) for krill oil. The analysis group of fatty acids was implemented according to Codex Standards for krill oil as follows: myristic acid (C14:0), palmitic acid (C16:0), palmitoleic acid (C16:1), oleic acid (C18:1), linoleic acid (C18:2, n-6), eicosenoic acid (C20:1, n-9), eicosapentaenoic acid (C20:5, n-3), and docosahexaenoic acid(C22:6, n-3). There was a significant difference in the content of myristic acid, palmitic acid, palmitoleic acid, linoleic acid, and eicosenoic acid between four krill oils (KO 2, 6, 8, and 15) and other samples. The concentrations of myristic acid, palmitic acid, and palmitoleic acid in the four krill oils were observed to be comparatively reduced in comparison to other krill oils, yet displayed analogous patterns to the Codex Standards for soybean and arachis oils [31, 44]. The ranges of linoleic acid and eicosenoic acid according to notified standards for krill oil are as follows: linoleic acid, not detected (ND)-3.0% (w/w); eicosenoic acid, not applicable or available (NA), whereas four krill oils indicated higher contents of two fatty acids than other KOs. These results were in line with the range of arachis oil according to Codex Standard [44]. However, numerous krill oil products promote their biochemical functionality without an authorized mark, leading to widespread consumer misperception regarding their status as functional foods. Therefore, a method for accurate and efficient analysis of authenticity and adulteration of krill oil supplements with vegetable oil such as soybean and peanut is urgently needed. Based on the results of this investigation, it was suggested to the Korean Ministry of Food and Drug Safety (MFDA) that standards of fatty acids composition for krill oil should be established. Accordingly, MFDA recently included linoleic acid (3% w/w or less) and myristic acid (5–13% w/w) among the standard fatty acid components for krill oil on September 30, 2021 [49]. Importantly, it is advisable to conduct consistent and frequent surveillance of krill oil supplements to guarantee their safety and to provide suitable guidance in Korea on the appropriate consumption to diverse consumer groups.

## Figures and Tables

**Table 1:** Acid value (AV) in krill oil supplements.

No. of samples	Acid value (AV) ^a^ (mg KOH/ g)	RSD ^b^	No. of samples	Acid value (AV) (mg KOH/ g)	RSD ^b^
1	13.0 ± 0.69	5.31	11	12.4 ± 0.59	4.76
2	20.9 ± 3.14	1.50	12	28.2 ± 0.02	0.07
3	19.0 ± 0.61	3.21	13	7.4 ± 0.26	3.51
4	12.1 ± 0.25	2.07	14	16.5± 0.41	2.48
5	25.1 ± 0.02	0.08	15	25.4 ± 3.35	1.32
6	25.4 ± 3.57	1.41	16	21.0 ± 0.09	0.43
7	43.7 ± 0.22	0.50	17	20.7 ± 0.55	2.66
8	20.7 ± 3.05	1.47	18	20.4 ± 0.26	1.27
9	17.0 ± 0.43	2.53	19	29.5 ± 1.70	5.76
10	27.4 ± 1.06	3.87	20	13.0 ± 0.37	2.85

^a^The average of triplicate acid value (AV) measurements

^b^Relative Standard Deviation (%RSD= Standard Deviation/ average × 100)

**Table 2-1:** Fatty acid (myristic acid, palmitic acid, palmitoleic acid, and oleic acid) analysis results of the 20 krill oil supplements.

No.	Myristic acid (C14:0) ^a^	RSD ^b^	Palmitic acid (C16:0) ^a^	RSD ^b^	Palmitoleic acid (C16:1) ^a^	RSD ^b^	Oleic acid (C18:1, n-9) ^a^	RSD ^b^
1	12.2	2.46	23.5	0.34	7.3	3.29	17.4	2.76
2	ND^c^	-	11.2	1.07	0.3	3.33	11.6	1.21
3	6.8	3.82	23.0	0.57	4.9	3.06	15.3	2.88
4	10.0	3.60	23.4	1.79	6.8	1.18	17.9	0.45
5	10.6	0.47	22.6	0.97	7.3	1.51	18.5	0.43
6	ND	-	10.8	0.56	0.3	3.33	15.5	0.26
7	7.4	0.41	20.6	0.92	6.7	1.49	16.5	0.48
8	ND	-	10.7	1.40	0.2	5.00	15.5	0.52
9	9.7	3.71	17.0	3.94	6.0	3.33	17.0	3.94
10	11.7	1.37	22.1	1.72	7.6	1.71	19.3	1.40
11	10.2	1.57	21.3	1.46	8.2	0.73	20.2	0.50
12	9.5	0.11	23.7	2.41	6.4	0.63	17.9	1.23
13	13.5	1.78	22.8	0.48	10.6	2.36	21.8	1.28
14	7.5	4.80	22.3	2.74	5.3	1.32	15.6	1.54
15	ND	-	10.5	1.81	0.2	2.50	15.4	1.56
16	8.4	2.62	20.7	1.30	6.8	1.32	18.7	1.66
17	10.0	1.20	22.6	1.24	6.0	0.83	18.3	0.87
18	6.9	3.48	22.0	5.73	4.5	1.78	14.7	2.99
19	10.4	0.96	23.8	2.35	7.3	0.55	20.0	1.10
20	8.9	4.27	22.5	1.87	6.4	2.81	18.2	4.78

^a^The average of triplicate measurements of the acid value(g/100 g; % (w/w))

^b^Relative Standard Deviation (%RSD= Standard Deviation/ average × 100)

^c^ND: not detected

**Table 3:** The typical fatty acid compositions of commercial krill oils, krill oil, and soybean oil.

Fatty acid ^a^	Commercial krill oils		Krill oil ^b^	Soybean oil ^c^	Arachis oil ^c^
	(16)	(4)			
Myristic acid(C14:0)	6.8 - 12	ND ^d^	5.0 - 13.0	ND - 0.2	ND – 0.1
Palmitic acid(C16:0)	17.0 - 23.8	10.5 – 11.2	17.0 - 24.6	8.0 - 13.5	8.0 – 14.0
Palmitoleic acid(C16:1)	4.5 - 10.6	0.2 – 0.3	2.5 - 9.0	ND - 0.2	ND – 0.2
Oleic acid(C18:1, n-9)	14.7 – 20.2	11.6 – 15.6	6.0 - 14.5	17.0 - 30.0	35.0 – 69.0
Linoleic acid(C18:2, n-6)	0.5 - 2.8	29.6 – 31.4	ND - 3.0	48.0 - 59.0	12.0 – 43.0
Eicosenoic acid(C20:1, n-9)	0.4 – 1.0	1.3 – 1.4	NA ^d^	ND - 0.3	0.7 – 1.7
Eicosapentaenoic acid(C20:5, n-3)	9.7 – 26.0	1.3 – 1.4	14.3 - 28.0	-^e^	-^e^
Docosahexaenoic acid(C22:6, n-3)	4.5 – 11.3	9.4 – 10.2	7.1 - 15.7	-^e^	-^e^

^a^Fatty acid profiles (g/100 g; % (w/w))

^b^Codex Standard 329-2017 (2017)

^c^Codex Standard 210-1999 (2015)

^d^NA: not applicable or available, ND: not detected, defined as ≤ 0.05%

^e^Not listed in the Codex Standard 210-1999 (2015)

**Table 2-2:** Fatty acid (linoleic acid, eicosenoic acid, eicosapentaenoic acid, and docosahexaenoic acid) analysis results of the 20 krill oil supplement.

No.	Linoleic acid (C18:2, n-6) ^a^	RSD ^b^	Eicosenoic acid (C20:1, n-9) ^a^	RSD ^b^	Eicosapentaenoic acid (C20:5, n-3) ^a^	RSD ^b^	Docosahexaenoic acid (C22:6, n-3) ^a^	RSD ^b^
1	0.5	4.00	0.8	2.50	14.4	0.97	6.8	1.18
2	31.4	1.18	0.9	3.89	16.2	2.41	10.2	2.25
3	1.2	3.33	0.4	2.50	23.6	2.88	11.1	2.97
4	2.7	0.74	0.6	5.00	17.9	0.45	8.0	4.25
5	2.8	0.36	0.6	1.67	15.2	0.39	7.5	0.40
6	29.6	0.57	1.3	2.31	15.5	0.26	9.5	0.32
7	2.0	1.00	0.7	1.43	26.0	1.12	8.8	1.25
8	29.9	1.10	1.3	0.77	15.5	0.52	9.4	2.77
9	2.3	3.91	0.6	3.33	18.1	4.48	8.7	0.57
10	2.8	1.07	0.6	3.33	14.2	2.11	7.2	1.94
11	2.4	0.83	1.0	2.00	12.5	2.08	8.7	1.84
12	2.4	0.83	0.6	1.67	17.9	1.23	8.0	1.88
13	2.5	0.80	0.9	1.11	9.7	4.85	4.5	2.67
14	2.5	0.80	0.4	5.13	22.5	1.56	10.3	1.07
15	30.0	1.80	1.3	3.85	15.4	1.56	9.8	0.41
16	4.2	0.95	0.9	2.22	21.0	1.90	8.2	2.44
17	2.3	0.87	0.7	1.43	15.3	0.52	7.9	0.38
18	2.3	3.91	0.4	2.50	23.3	4.55	10.5	4.10
19	2.3	3.04	0.9	1.11	15.5	1.81	6.6	1.36
20	2.5	4.40	0.5	4.20	15.1	5.30	8.1	4.81

^a^The average of triplicate measurements of the acid value (g/100 g; % (w/w))

^b^Relative Standard Deviation (%RSD= Standard Deviation/ average × 100)
